# Comparação dos Valores de Pressão Arterial e dos Medicamentos Anti-Hipertensivos Utilizados por Brasileiros não Afrodescendentes e Afrodescendentes com Hipertensão

**DOI:** 10.36660/abc.20250316

**Published:** 2026-01-26

**Authors:** Maicon Borges Euzébio, Priscila Valverde de Oliveira Vitorino, Andrea A. Brandão, Eduardo Costa Duarte Barbosa, Celso Amodeo, Audes Feitosa, Marcus Vinicius Bolivar Malachias, Marco Antonio MotaGomes, Rui Manoel dos Santos Póvoa, Renato Delascio Lopes, Paulo Cesar B Veiga Jardim, Ana Luiza Lima Souza, Antonio Coca, Weimar Kunz Sebba Barroso

**Affiliations:** 1 Universidade Federal de Goiás Goiânia GO Brasil Universidade Federal de Goiás, Goiânia, GO – Brasil; 2 Instituto Federal de Goiás Águas Lindas de Goiás GO Brasil Instituto Federal de Goiás, Águas Lindas de Goiás, GO – Brasil; 3 Pontifícia Universidade Católica de Goiás Goiânia GO Brasil Pontifícia Universidade Católica de Goiás (PUC Goiás), Goiânia, GO – Brasil; 4 Universidade do Estado do Rio de Janeiro Rio de Janeiro RJ Brasil Universidade do Estado do Rio de Janeiro, Rio de Janeiro, RJ – Brasil; 5 Complexo Hospitalar Santa Casa de Misericórdia de Porto Alegre Porto Alegre RS Brasil Complexo Hospitalar Santa Casa de Misericórdia de Porto Alegre, Porto Alegre, RS – Brasil; 6 Escola Paulista de Medicina Universidade Federal de São Paulo São Paulo SP Brasil Escola Paulista de Medicina da Universidade Federal de São Paulo (EPM/UNIFESP), São Paulo, SP – Brasil; 7 Universidade de Pernambuco Recife PE Brasil Universidade de Pernambuco, Recife, PE – Brasil; 8 Universidade Católica de Pernambuco Recife PE Brasil Universidade Católica de Pernambuco, Recife, PE – Brasil; 9 Faculdade de Ciências Médicas de Minas Gerais Belo Horizonte MG Brasil Faculdade de Ciências Médicas de Minas Gerais, Belo Horizonte, MG – Brasil; 10 Instituto de Hipertensão Arterial Diretoria Clínica Horizonte MG Brasil Instituto de Hipertensão Arterial – Diretoria Clínica, Horizonte, MG – Brasil; 11 Centro Universitário CESMAC Centro de Pesquisas Clínicas Dr. Marco Mota Maceió AP Brasil Centro Universitário CESMAC – Centro de Pesquisas Clínicas Dr. Marco Mota, Maceió, AP – Brasil; 12 Universidade de São Paulo Faculdade de Medicina São Paulo SP Brasil Universidade de São Paulo Faculdade de Medicina, São Paulo, SP – Brasil; 13 Duke University Hospital Durham North Carolina EUA Duke University Hospital, Durham, North Carolina – EUA; 14 Hypertension and Vascular Risk Unit Hospital Clinic University of Barcelona Barcelona Espanha Hypertension and Vascular Risk Unit. Hospital Clinic. University of Barcelona, Barcelona – Espanha

**Keywords:** Hipertensão, Fatores Raciais, Características da População, Anti-Hipertensivos

## Abstract

**Fundamento:**

A prevalência da hipertensão (HAS) é elevada em comunidades étnicas, particularmente entre adultos afrodescendentes nos Estados Unidos. Além disso, há uma escassez de estudos que abordem essa questão na população afrodescendente brasileira.

**Objetivos:**

Analisar o controle da pressão arterial e o uso de medicamentos anti-hipertensivos entre brasileiros afrodescendentes e não afrodescendentes.

**Métodos:**

Este estudo transversal avaliou dados do Primeiro Registro Brasileiro de Hipertensão, que incluiu indivíduos com mais de 18 anos de idade, autodeclarados como afrodescendentes ou não afrodescendentes, com HAS há pelo menos quatro semanas ou em uso de medicamento anti-hipertensivo. As comparações foram realizadas utilizando testes t ou o teste de Mann-Whitney. Foi adotado nível de significância de p < 0,05.

**Resultados:**

Um total de 2.643 participantes foi incluído, dos quais 82,8% eram não afrodescendentes e 17,1% eram afrodescendentes. As taxas de HAS não controlada foram de 44,68% entre os não afrodescendentes e de 54,64% entre os afrodescendentes. Os valores medianos da pressão arterial sistólica (PAS), pressão arterial diastólica (PAD) e índice de massa corporal (IMC) foram mais elevados nos afrodescendentes em comparação aos não afrodescendentes (p < 0,001). A distribuição das classes de medicamentos anti-hipertensivos variou entre as populações. Não foram observadas diferenças significativas no controle da HAS entre os usuários de betabloqueadores nos grupos afrodescendentes e não afrodescendentes, mesmo quando estratificados por sexo.

**Conclusão:**

A população afrodescendente no Brasil apresentou maior prevalência de HAS não controlada e valores mais elevados de PAS, PAD e IMC em comparação à população não afrodescendente. A escolha dos medicamentos anti-hipertensivos diferiu entre os grupos, sendo os tiazídicos mais comumente prescritos para afrodescendentes e os bloqueadores dos receptores de angiotensina II para não afrodescendentes. No entanto, não foram observadas diferenças significativas no controle da PA entre os grupos que utilizavam betabloqueadores, independentemente do sexo.

## Graphical abstract

**Figura Central:**
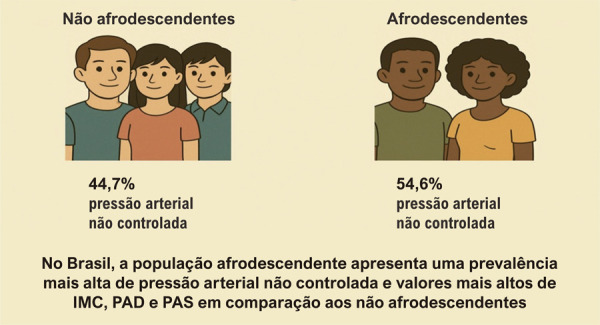
Comparação dos Valores de Pressão Arterial e dos Medicamentos Anti-Hipertensivos Utilizados por Brasileiros não Afrodescendentes e Afrodescendentes com Hipertensão

Indivíduos que se auto declararam “pretos” foram considerados afrodescendentes, e aqueles que se auto declararam “brancos”, “pardos” (raça mista) ou “asiáticos” foram considerados não afrodescendentes. IMC: índice de massa corporal; PAD: pressão arterial diastólica; PAS: pressão arterial sistólica.

## Introdução

A hipertensão (HAS) é o principal fator de risco modificável para doenças cardiovasculares (DCV) em todo o mundo e continua sendo a principal causa de morte nas Américas.^1^ Nos Estados Unidos, comunidades étnicas como afrodescendentes, hispânicos e asiáticos tendem a desenvolver HAS mais precocemente e apresentam menores taxas de controle da pressão arterial (PA) em comparação com brancos não hispânicos.^2^ O conceito de raça é amplamente debatido e, em estudos epidemiológicos, pode obscurecer as verdadeiras causas das disparidades em saúde entre grupos raciais — desviando a atenção de fatores socioeconômicos e ambientais — o que contribui para as menores taxas de controle da HAS observadas nessas comunidades.^3 , 4^

Globalmente, em 2019, as taxas de diagnóstico de HAS foram de 59% entre mulheres e 49% entre homens, enquanto as taxas de tratamento foram de 47% nas mulheres e 38% nos homens.^5^ Ao longo das últimas quatro décadas, os padrões globais de HAS se modificaram: a prevalência diminuiu em países de alta renda, enquanto aumentou simultaneamente em nações de baixa renda no Sul da Ásia e na África Subsaariana. Enquanto isso, o número médio de casos por ano permaneceu inalterado na Europa Central e Oriental.^6^

A prevalência da HAS é significativa em comunidades étnicas, especialmente entre adultos afrodescendentes. A taxa de mortalidade associada à HAS entre homens e mulheres de ascendência africana é aproximadamente duas vezes maior em comparação com outros grupos raciais/étnicos. As origens dessas disparidades são multifatoriais e ainda não totalmente elucidadas.^2^ Manter o controle da HAS continua sendo um desafio persistente tanto na prática clínica quanto na saúde pública.^7^

Apesar da existência de disparidades socioeconômicas e raciais em saúde no Brasil, as implicações da segregação nos desfechos de saúde ainda não foram adequadamente analisadas nesse contexto. Isso torna o Brasil — com suas semelhanças e diferenças em relação aos Estados Unidos — um cenário particularmente relevante para investigações nessa área.^8^

No Brasil, a fusão de afrodescendentes e grupos de pessoas pardas forma uma ampla categoria populacional conhecida como “negros”, com estimativas sugerindo que o número de pessoas com ascendência africana excede a simples soma de afrodescendentes e pardos.^9^ Atualmente, 55,5% da população brasileira se identifica como negra, sendo 20,7 milhões (10,3%) autodeclarados afrodescendentes e 92,1 milhões (45,3%) pardos. Os brancos totalizam 88,3 milhões (43,5%), os indígenas 1,7 milhão (0,6%) e os asiáticos 850 mil (0,4%).^10^

Considerando que uma parcela significativa da população brasileira possui ascendência africana, e que o menor controle da HAS é observado especificamente nessa comunidade étnica — evidenciando os desafios enfrentados pelo Estado — compreender tais disparidades pode contribuir para o desenvolvimento de políticas públicas mais justas e para aprimorar a efetividade do cuidado clínico voltado a essas populações vulneráveis.^11^

## Métodos

Este estudo transversal utilizou dados do Primeiro Registro Brasileiro de Hipertensão (1RBH),^12^ aprovado pelo Comitê de Ética em 17 de fevereiro de 2014, sob o protocolo nº 532.146. O 1RBH foi um estudo multicêntrico que analisou pacientes diagnosticados com HAS. Incluiu participantes de todas as regiões do Brasil, provenientes de serviços de saúde públicos, privados e mistos. A coleta de dados ocorreu entre junho de 2013 e outubro de 2015.

O registro foi conduzido em conformidade com diretrizes nacionais e internacionais, incluindo a Declaração de Helsinque, a Resolução CNS 196/96 e seus complementos (CNS/MS), as Diretrizes de Boas Práticas Clínicas da ICH (1996), o Documento das Américas (2005) e a Resolução 466/2012. Cada centro de pesquisa clínica submeteu o protocolo do estudo, o Termo de Consentimento Livre e Esclarecido (TCLE) e toda a documentação pertinente ao Comitê de Ética em Pesquisa (CEP) de sua instituição para revisão e aprovação antes de iniciar quaisquer procedimentos incluídos no registro.

Após a obtenção do consentimento livre e esclarecido, os prontuários médicos foram revisados e os participantes foram entrevistados em cada centro por pesquisadores treinados para o preenchimento de um formulário eletrônico de relato de caso.

A análise da frequência absoluta e da distribuição percentual das classes de medicamentos anti-hipertensivos utilizadas pela população foi realizada considerando as seguintes categorias: bloqueadores dos receptores de angiotensina (BRAs), diuréticos tiazídicos, betabloqueadores (BBs), inibidores da enzima conversora de angiotensina (IECA), bloqueadores dos canais de cálcio (BCCs) e diuréticos de alça. Indivíduos que se autodeclararam pretos foram classificados como afrodescendentes, enquanto aqueles que se autodeclararam brancos, pardos (mestiços) ou asiáticos foram classificados como não afrodescendentes.

Seguindo as recomendações das diretrizes vigentes à época do estudo, a média de duas medidas periféricas de PA, realizadas durante a visita inicial, foi utilizada para avaliar o controle da PA nos grupos. Os participantes foram classificados como tendo PA não controlada se a PA sistólica (PAS) fosse ≥ 140 mmHg, a PA diastólica (PAD) fosse ≥ 90 mmHg, ou ambas. Aqueles com PA controlada apresentavam PAS < 140 mmHg e PAD < 90 mmHg.

Os critérios de inclusão foram: assinatura do Termo de Consentimento Livre e Esclarecido (TCLE), idade mínima de 18 anos e diagnóstico confirmado de HAS por pelo menos quatro semanas, com PAS ≥ 140 mmHg e/ou PAD ≥ 90 mmHg medida na posição sentada,^12^ ou uso atual de medicação anti-hipertensiva, além da matrícula regular no centro/instituição participante.

Foram excluídos indivíduos com insuficiência renal em diálise, hospitalização no momento da inclusão ou nos 30 dias anteriores, instabilidade hemodinâmica que exigisse o uso de drogas vasoativas nos últimos 30 dias, insuficiência cardíaca classificada como classe funcional III ou IV, gestantes e/ou lactantes, doença hepática grave, transtornos psiquiátricos que impedissem a adesão ao protocolo, histórico de acidente vascular cerebral ou infarto do miocárdio nos 30 dias anteriores à inclusão no estudo, doenças graves avaliadas pelo investigador e neoplasias com prognóstico de sobrevida inferior a um ano.

### Análise estatística

As variáveis contínuas foram descritas por meio de média e desvio padrão ou mediana (intervalo interquartil), dependendo da normalidade dos dados, a qual foi testada pelo teste de Shapiro–Wilk. As variáveis categóricas foram apresentadas em números absolutos e percentuais. As análises estatísticas foram conduzidas em subgrupos predefinidos, considerando-se valores de p < 0,05 como estatisticamente significativos. As análises comparativas foram realizadas pelo teste de Mann–Whitney quando p < 0,05 e pelo teste t quando p > 0,05. Para a análise de regressão logística binária, foi aplicado intervalo de confiança de 95%. Nesse contexto, PA não controlada, não uso de BBs e sexo feminino foram definidos como nível de referência 1 para avaliar se o uso de BBs poderia estar associado a melhor controle da PA. As análises estatísticas foram realizadas utilizando o software Jamovi, versão 2.3.

## Resultados

### Características dos pacientes

O estudo incluiu 2643 participantes de 45 centros de pesquisa distribuídos por todas as regiões do Brasil, dos quais 2191 (82,9%) se autodeclararam não afrodescendentes e 452 (17,1%) se autodeclararam afrodescendentes. Os pacientes foram atendidos em unidades de saúde públicas (46,7%), privadas (31,1%) e mistas (22,2%). Informações detalhadas sobre idade, Índice de Massa Corporal (IMC), Circunferência da Cintura (CC) e sexo da população geral, bem como dos grupos não afrodescendentes e afrodescendentes, estão disponíveis na Tabela 1 .

**Tabela 1:** – Características dos pacientes incluídos no estudo entre 2013 e 2015

Descrição	n	Não afrodescendentes	Afrodescendentes	p
Etnia autodeclarada	2643	2191 (82,9%)	452 (17,1%)	
Sexo feminino	1472 (55,6%)	1212 (55,3%)	260 (57,5%)	0,390
Idade, anos	62,0 (54,1 - 69,4)	61,9 (54,1 - 69,4)	62,5 (54,2 - 69,7)	0,922
CC, cm	98,0 (90,0 - 107)	98,3 (90,0 - 107)	98,0 (91,0 - 106)	0,788
IMC, Kg/m ^2^	28,7 (25,6 - 32,0)	28,4 (25,5 - 31,9)	29,4 (26,0 - 32,9)	0,002
HAS, duração (anos)	10 (4 - 20)	10 (5 - 20)	10 (5 - 20)	0,044
HAS tratamento, anos	10 (4 - 17)	10 (4 - 17)	10 (5 - 20)	0,072
Diabetes Mellitus (DM)	784 (29,66%)	656 (29,9%)	128 (28,3%)	0,492
DM, duração (anos)	5,1 (3 - 14)	7 (3 - 15)	6 (2 -10)	0,007
Consumo de bebida alcoólica	197 (7,45%)	153 (6,9%)	44 (9,7%)	0,043
Tabagismo	165 (6,24%)	134 (6,1%)	31 (6,8%)	0,552

DM: Diabetes Mellitus; HAS: hipertensão; IMC: índice de massa corporal; CC: circunferência da cintura. Fonte: elaborado pelo autor.

### Valores de pressão arterial periférica

Os valores da PAS e PAD periférica foram mais elevados entre os indivíduos afrodescendentes em comparação com os não afrodescendentes ( Tabela 2 ).

**Tabela 2:** – Análise comparativa dos valores de pressão arterial periférica em populações brasileiras hipertensas não afrodescendentes e afrodescendentes (2013–2015)

Valores de pressão arterial	n (não afrodescendentes; não afrodescendentes	Não afrodescendentes	Afrodescendentes	p
PAS	2191; 452	132 (120–150)	140 (129–151^)^	< 0,001*
PAD	2191; 452	80,0 (73,5–90,0)	85,5 (80,0–93,0)	< 0,001*

* teste de Mann-Whitney, se p < 0,05. PAD: pressão arterial diastólica medida na visita inicial; PAS: pressão arterial sistólica medida na visita inicial; os valores de PAS e PAD são apresentados em mmHg. Fonte: elaborado pelo autor.

Avaliação do controle da pressão arterial em populações brasileiras hipertensas não afrodescendentes e afrodescendentes

**Figura 1:**
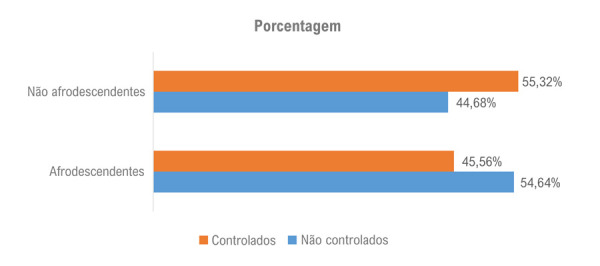
– Percentual de indivíduos não afrodescendentes e afrodescendentes com pressão arterial controlada e não controlada, incluídos no estudo entre 2013 e 2015.

A população afrodescendente apresentou uma maior porcentagem de PA não controlada em comparação com a população não afrodescendente ( Figura 1 e Figura Central ).

Medicamentos anti-hipertensivos utilizados por populações brasileiras hipertensas afrodescendentes e não afrodescendentes

Proporcionalmente, a população afrodescendente utilizou um maior número de classes de medicamentos anti-hipertensivos em comparação com a população não afrodescendente ( Tabela 3 ).

**Tabela 3:** – Distribuição da frequência absoluta e do percentual das classes de medicamentos anti-hipertensivos utilizados por populações brasileiras hipertensas não negras e negras, incluídas no estudo entre 2013 e 2015

Número de classes *	Não afrodescendentes (n = 2191)	Afrodescendentes (n = 452)	p
0	109 (5,00%)	16 (3,50%)	0,967*
1	478 (21,80%)	115 (25,40%)	
2	793 (36,20%)	133 (29,40%)	
3	550 (25,10%)	120 (26,50%)	
4	204 (9,30%)	47 (10,40%)	
5	46 (2,10%)	17 (3,80%)	
6	11 (0,50%)	4 (0,90%)	

* teste de Mann-Whitney, se p < 0,05.

Classes de medicamentos anti-hipertensivos utilizados por indivíduos hipertensos no Brasil, nas populações afrodescendentes e não afrodescendentes

A distribuição percentual das três primeiras classes de medicamentos anti-hipertensivos foi diferente entre os grupos afrodescendentes e não afrodescendentes, tornando-se semelhante apenas a partir da quarta classe ( Tabela 4 ).

**Tabela 4:** – Ordem decrescente do percentual das classes de medicamentos anti-hipertensivos utilizados por populações brasileiras hipertensas, não afrodescendentes e afrodescendentes, incluídas no estudo entre 2013 e 2015

Ordem	Não afrodescendentes (%) (n = 2,191)	Afrodescendentes (%) (n = 452)
1º	47,6% (BRA)	52% (Tiazídicos)
2º	47,3% (Tiazídicos)	38,3% (Betabloqueadores)
3º	42,4% (Betabloqueadores)	38,1% (BRA)
4º	33,5% (IECA)	35,6% (IECA)
5º	28,1% (BCCs)	35,0% (BCCs)
6º	10,6% (Diuréticos de alça)	16,6% (Diuréticos de alça)
7º	7,4% (Espironolactona)	9,7% (Espironolactona)

BRA: bloqueadores dos receptores de angiotensina; BCCs: bloqueadores dos canais de cálcio; Tiazídicos: diuréticos tiazídicos; IECA: inibidores da enzima conversora de angiotensina.

Associação entre o controle da pressão arterial e o uso de betabloqueadores em indivíduos brasileiros hipertensos afrodescendentes e não afrodescendentes, considerando o sexo

Não foram encontradas diferenças significativas no controle da PA entre os grupos afrodescendentes e não afrodescendentes que utilizaram BBs, independentemente do sexo ( Tabelas 5 e 6 ).

**Tabela 5:** – Análises de regressão logística binária mostrando a razão de chances (OR) e o intervalo de confiança de 95% (IC) para perfis de pressão arterial controlada vs. não controlada, e para indivíduos do sexo feminino e masculino que utilizam betabloqueadores na população brasileira preta hipertensa incluída no estudo entre 2013 e 2015

Variáveis independentes	OR	IC (95%)	p
Intercepto	0,61	0,44 – 0,84	0,003
Betabloqueador			
Usa	1,04	0,70 - 1,53	0,840
Não usa	1		
Sexo			
Masculino	1,25	0,85 – 1,83	0,254
Feminino	1		

As estimativas representam o logaritmo das chances de “perfil de PA = Controlado” vs. “perfil de PA = Não controlado”.

**Tabela 6:** – Análises de regressão logística binária mostrando a razão de chances (OR) e o Intervalo de Confiança de 95% (IC) para perfis de pressão arterial controlada vs. não controlada, e para indivíduos do sexo feminino e masculino que utilizam betabloqueadores na população brasileira não preta hipertensa incluída no estudo entre 2013 e 2015

Variáveis independentes	OR	IC (95%)	p
Intercepto	1,16	1,00 – 1,36	0,039
Betabloqueador			
Usa	0,93	0,78 – 1,11	0,433
Não usa	1		
Sexo			
Masculino	0,87	0,74 – 1,04	0,133
Feminino	1		

As estimativas representam o logaritmo das chances de “perfil de PA = Controlado” vs. “perfil de PA = Não controlado”

## Discussão

Este estudo mostra que a prevalência de HAS não controlada foi maior entre indivíduos brasileiros afrodescendentes, que também apresentaram valores mais elevados de PAS, PAD e IMC em comparação com a população não afrodescendente. Observamos diferenças na seleção dos medicamentos anti-hipertensivos prescritos pelos médicos, com maior uso de diuréticos tiazídicos entre os indivíduos afrodescendentes. No entanto, entre aqueles também tratados com BBs, não foram identificadas diferenças significativas no controle da HAS entre os grupos afrodescendentes e não afrodescendentes, independentemente do sexo.

De acordo com o último censo brasileiro, indivíduos autodeclarados afrodescendentes representam 10,2% da população brasileira,^10^ o que reforça a relevância do nosso estudo, já que alcançamos 17,1% de representatividade na amostra.

Um estudo recente realizado no Brasil revelou que a probabilidade de desenvolver HAS aumentava com a idade (particularmente entre indivíduos afrodescendentes) e era menor na região Norte do país, independentemente da faixa etária ou do sexo. Observou-se maior probabilidade de desenvolver HAS entre mulheres com menor nível educacional e estilo de vida sedentário, bem como entre homens que vivem com suas esposas e apresentam baixos níveis de atividade física.^13^

Nosso estudo também analisou a questão do alcoolismo e, com significância estatística, corroborou os achados de outros estudos semelhantes, os quais indicam que, no Brasil, a maior taxa de incidência dessa condição foi novamente observada entre a população afrodescendente. Entre 2013 e 2019, a prevalência aumentou entre afrodescendentes (de 16,6% para 19,6%), pardos (de 11,2% para 17,5%) e brancos (de 12,4% para 16,0%), enquanto diminuiu entre asiáticos (de 14,4% para 12,7%).^14^

Alguns estudos apontaram maior prevalência de diabetes tipo 2 e intolerância à glicose entre pessoas afrodescendentes em comparação aos brancos.^15 , 16^ Além disso, um estudo brasileiro mostrou que mulheres obesas de pele mais escura apresentaram maior associação com diabetes e intolerância à glicose, independentemente da idade e do IMC.^17^

Curiosamente, nossos dados revelaram uma prevalência quase idêntica de diabetes entre as populações; entretanto, a população não afrodescendente apresentou um tempo maior até o diagnóstico. Isso pode sugerir que o acesso mais fácil aos serviços de saúde entre os indivíduos não afrodescendentes favoreceu o diagnóstico precoce e um manejo mais eficaz da doença, o que, por sua vez, pode ter ajudado a mitigar os efeitos nocivos do diabetes na progressão da HAS.

Os participantes afrodescendentes apresentaram medianas significativamente mais elevadas tanto de PAS quanto de PAD em comparação aos não afrodescendentes, destacando uma tendência de valores mais altos de PA nesse grupo. Essa disparidade pode estar relacionada a fatores socioeconômicos e comportamentais, como diferenças no acesso aos serviços de saúde, condições de vida, educação em saúde e a influência de determinantes sociais que afetam desproporcionalmente a população afrodescendente, contribuindo para um controle menos eficaz da HAS.

No estudo de coorte ELSA-Brasil, ao analisar a incidência de HAS por sexo e raça, os homens afrodescendentes apresentaram a maior incidência (59,4 por 1.000 pessoas-ano), enquanto as mulheres brancas tiveram a menor incidência (30,5 por 1.000 pessoas-ano). Após o ajuste por idade e histórico familiar, a taxa de incidência permaneceu significativamente mais elevada entre os homens afrodescendentes (2,25; IC 95%, 1,65–3,08), seguida por homens pardos, mulheres afrodescendentes, mulheres pardas e homens brancos, em comparação às mulheres brancas.^18^

Outros estudos também demonstraram níveis mais elevados de PAD entre populações afrodescendentes em comparação às não afrodescendentes.^19 - 22^ Além disso, pesquisas genéticas evidenciaram o papel de fatores biológicos na maior prevalência de HAS em populações de ascendência africana.^2 3^ No entanto, os fatores genéticos parecem exercer menor influência na população brasileira hipertensa afrodescendente, considerando que o efeito das condições socioeconômicas sobre o manejo da HAS tem sido mais evidente.

Estudos recentes sobre variações no comportamento da PA entre populações não afrodescendentes e afrodescendentes levantaram questões sobre a necessidade de estabelecer limites específicos de PA de acordo com a raça, o que pode melhorar a estimativa de risco e otimizar o manejo da HAS com o objetivo de reduzir disparidades étnicas.^22^ No Brasil, a população hipertensa afrodescendente apresentou uma taxa de controle 9,76% menor em comparação à população não afrodescendente. Essa diferença levanta questões importantes sobre os fatores que podem contribuir para a discrepância no controle da HAS.

Um estudo realizado nos Estados Unidos, utilizando critérios diagnósticos semelhantes para HAS (PAS ≥ 140 mmHg e/ou PAD ≥ 90 mmHg), avaliou uma amostra de 213.836 pacientes. A idade média dos participantes foi de 63,1 anos, sendo 55,5% mulheres e 70,8% brancos. O estudo constatou que, sem estratificação por cor autodeclarada, 29,7% dos pacientes apresentavam HAS não controlada.^24^

Outro estudo realizado em Londres investigou as disparidades no tratamento e no controle da PA em uma grande coorte de pacientes adultos com HAS (n = 156.290). O estudo revelou que o grupo étnico afrodescendente tinha menor probabilidade de alcançar o controle da PA em comparação ao grupo étnico branco, concluindo que indivíduos afrodescendentes e pessoas mais jovens são menos propensos a atingir o controle da HAS.^25^

Um estudo realizado nos Estados Unidos, envolvendo mais de 700.000 pacientes, relatou taxas anuais de HAS não controlada variando de 21,2% a 24,2%, sendo essa variação mais comum entre os homens. Os afro-americanos apresentaram a maior taxa (31,3%), enquanto os homens brancos registraram 19,4%. Entre as mulheres, as afro-americanas mostraram 28,6% de HAS não controlada, em comparação com 19,2% entre as mulheres brancas.^26^

Paralelamente, um estudo global que avaliou o controle da PA mostrou que a Coreia do Sul, o Canadá e a Islândia apresentaram as maiores taxas de tratamento e controle, com mais de 70% dos casos recebendo tratamento e mais de 50% alcançando controle. Da mesma forma, avanços significativos foram observados em países de alta renda e em alguns países recentemente elevados à categoria de renda média-alta, incluindo Costa Rica, Taiwan, Cazaquistão, África do Sul, Brasil, Chile, Turquia e Irã.^1^

Um estudo realizado nos Estados Unidos investigou variações no controle da PA entre pacientes afrodescendentes e brancos antes e depois da implementação de um programa de melhoria da qualidade. O estudo observou que as disparidades no controle da PA entre pacientes afrodescendentes e brancos diminuíram, mas não foram totalmente eliminadas, mesmo após a introdução de estratégias de melhoria da qualidade voltadas para a redução dessas disparidades.^27^

O Registro Nacional de Controle da Hipertensão no Brasil, ao avaliar a HAS por meio de medidas realizadas em consultório e pelo monitoramento residencial da PA, sem estratificação por raça, revelou que o controle da PA em consultórios, entre uma amostra de brasileiros, foi de 56,3%. Esse percentual aumentou para 61% quando a PA foi medida em casa e foi de 46,4% quando o controle foi observado tanto no consultório quanto em casa.^28^

Nos Estados Unidos, as taxas de controle da PA têm diminuído na última década, particularmente entre grupos raciais e étnicos. Afro-americanos não hispânicos apresentaram taxas de controle 10% menores do que seus pares brancos não hispânicos.^29^ Uma análise recente das taxas de controle da HAS pelo *National Health and Nutrition Examination Survey* (NHANES) revelou que as taxas de controle da PA foram menores entre indivíduos hispânicos (40%), afrodescendentes não hispânicos (39%) e asiático-americanos (38%), em comparação com indivíduos brancos não hispânicos (49%).^30^

Os afro-americanos nos Estados Unidos apresentam taxas de prevalência consideravelmente mais altas e menores taxas de controle da HAS em comparação com as populações brancas.^31 , 32^ Além disso, a frequente sub-representação de afro-americanos em ensaios clínicos cardiovasculares limita a aplicação segura dos resultados de diversos estudos nessa população específica.^33^

Vários estudos têm destacado disparidades étnicas no controle da HAS, particularmente entre afro-americanos e afro-caribenhos (especialmente entre homens afro-americanos), e têm demonstrado a necessidade urgente de intervenções direcionadas para enfrentar de forma eficaz tais inconsistências no manejo da HAS.^24 - 26^

Embora em nosso estudo o número mais comum de classes de medicamentos tenha sido semelhante em ambos os grupos, foram observadas diferenças nas proporções de uso. Por exemplo, os afrodescendentes utilizaram monoterapia com maior frequência (25,4%) em comparação aos não afrodescendentes (21,8%). Em contraste, os indivíduos brancos, proporcionalmente, utilizaram dois medicamentos anti-hipertensivos mais frequentemente do que os afrodescendentes. Finalmente, a população afrodescendente, proporcionalmente, utilizou três ou mais classes de medicamentos com maior frequência do que a população não afrodescendente.

Em uma coorte prospectiva de mulheres norte-americanas (n = 3302; idade entre 42 e 52 anos), verificou-se que as mulheres afrodescendentes também apresentaram maior probabilidade de utilizar mais de duas classes de medicamentos anti-hipertensivos (OR, 1,95; IC 95%, 1,55–2,45) em comparação às mulheres brancas.^34^

Um estudo realizado nos Estados Unidos envolvendo 23825 adultos com idade ≥18 anos, que investigou disparidades raciais e étnicas no uso de medicamentos anti-hipertensivos entre adultos hipertensos de 2011 a 2018, revelou que a população afrodescendente tinha probabilidade significativamente maior de receber terapia combinada e medicamentos de combinação em comprimido único.^35^ Em nosso estudo, a população afrodescendente, em termos de frequência e sem diferença estatisticamente significativa na análise comparativa global, apresentou maior proporção de indivíduos em monoterapia.

Nosso estudo revelou que, entre os participantes afrodescendentes, os diuréticos tiazídicos foram a escolha mais comum (52%), seguidos pelos BB e pelos BRAs. Entre os participantes não afrodescendentes, os BRA foram os mais prescritos, seguidos pelos diuréticos tiazídicos e pelos BBs.

Uma revisão sistemática sobre padrões de prescrição de medicamentos nos Estados Unidos para indivíduos não afrodescendentes indicou que os medicamentos anti-hipertensivos mais comumente prescritos como monoterapia para adultos com HAS sem comorbidades foram os IECAs ou os BRA, seguidos pelos BCCs e pelos BB. As combinações duplas mais prevalentes foram diuréticos tiazídicos com IECA ou BRA, BB com BCC e BCC com IECA ou BRA.^36^ Outra revisão indicou que os diuréticos tiazídicos foram identificados como os anti-hipertensivos mais frequentemente prescritos em monoterapia, enquanto os IECA ou BRA foram os mais prescritos em outro estudo.^37^

Os resultados do Estudo ELSA-Brasil sugerem que, em uma amostra de adultos brasileiros em uso de monoterapia anti-hipertensiva, as diferenças no controle da PA entre diversos grupos raciais não são explicadas pela eficácia potencialmente menor dos IECAs e dos BRAs em indivíduos afrodescendentes. Ressalta-se que a população afrodescendente no Brasil apresenta características distintas e que as recomendações devem ser adaptadas ao contexto local, indicando que o menor controle da PA observado em indivíduos afrodescendentes pode estar mais relacionado aos determinantes sociais do que à classe de medicamento anti-hipertensivo utilizada.^38^

Esses resultados destacam a importância de considerar fatores étnicos e individuais na prescrição de medicamentos anti-hipertensivos, além de questões socioeconômicas, já que diferentes grupos étnicos podem apresentar respostas variadas aos tratamentos.^39^ Além disso, é evidente que existem diferenças nas classes de medicamentos prescritos no Brasil em comparação com os Estados Unidos. Neste estudo, também exploramos a hipótese de que o uso de BBs poderia estar associado a um melhor controle da HAS. No entanto, não identificamos diferenças significativas no controle da HAS entre os dois grupos, independentemente do sexo, o que nos levou a questionar se o uso de BBs em combinação poderia ter um efeito menor na redução da PA em comparação à monoterapia em nossas populações afrodescendentes e não afrodescendentes.

Uma meta-análise que avaliou 20 estudos revelou que os BBs, exceto o atenolol, são eficazes na redução da PA, tanto como adjuvantes da monoterapia quanto como parte de uma terapia anti-hipertensiva combinada. Não foram encontradas diferenças significativas nos efeitos de redução da PA entre combinações com e sem β-bloqueadores (PAS: –1,3 mmHg [–5,8 a 3,2]; PAD: –0,3 mmHg [–2,7 a 2,1]).^40^

Em outro estudo envolvendo 11860 participantes, os BBs de segunda e terceira geração reduziram a PA média em 1,75 mmHg (intervalo de confiança de 95%: 1,16–2,33; p < 0,001) em todos os participantes analisados, e em 1,93 mmHg (IC 95%: 0,86–3,00; p < 0,001) especificamente entre africanos hipertensos.^41^ Compreender por que o uso de β-bloqueadores não esteve associado a um melhor controle da PA permanece incerto. No entanto, isso pode estar relacionado ao fato de que muitos indivíduos hipertensos estavam utilizando mais de duas classes de medicamentos.

### Limitações

É importante observar que a análise não forneceu informações sobre as razões subjacentes às disparidades sociais, educacionais, econômicas e comportamentais — tais como acesso aos serviços de saúde, adesão ao tratamento ou fatores genéticos — o que pode limitar a interpretação dos resultados.

## Conclusões

No que diz respeito ao controle da PA, brasileiros afrodescendentes autodeclarados apresentaram maior proporção de HAS não controlada em comparação aos indivíduos não afrodescendentes. A população afrodescendente também apresentou valores mais elevados de PA periférica do que a população não afrodescendente. Foram observadas diferenças nas porcentagens dos medicamentos mais comumente utilizados e na distribuição das classes. Não foram encontradas associações significativas no controle da PA entre os grupos que utilizavam BBs, independentemente do sexo.
