# Supplementary material for: Comparação dos Valores de Pressão Arterial e dos Medicamentos Anti-Hipertensivos Utilizados por Brasileiros não Afrodescendentes e Afrodescendentes com Hipertensão
Source: Arq Bras Cardiol. 2026 Jan 26;122(12):e20250316. [Article in Portuguese] doi: 10.36660/abc.20250316 (PMC12978371; doi:10.36660/abc.20250316)
Supplement: Translation

# Translation: Comparison of Blood Pressure Values and Antihypertensive Medications Used in Non-Afro-Descendant and Afro-Descendant Brazilians with Hypertension

**DOI:** 10.36660/abc.20250316i

**Authors:** Maicon Borges Euzébio, Priscila Valverde de Oliveira Vitorino, Andrea A. Brandão, Eduardo Costa Duarte Barbosa, Celso Amodeo, Audes Feitosa, Marcus Vinicius Bolivar Malachias, Marco Antonio MotaGomes, Rui Manoel dos Santos Póvoa, Renato Delascio Lopes, Paulo Cesar B Veiga Jardim, Ana Luiza Lima Souza, Antonio Coca, Weimar Kunz Sebba Barroso

**Affiliations:** 1 Brazil Universidade Federal de Goiás, Goiânia, GO – Brazil; 2 Brazil Instituto Federal de Goiás, Águas Lindas de Goiás, GO – Brazil; 3 Brazil Pontifícia Universidade Católica de Goiás (PUC Goiás), Goiânia, GO – Brazil; 4 Brazil Universidade do Estado do Rio de Janeiro, Rio de Janeiro, RJ – Brazil; 5 Brazil Complexo Hospitalar Santa Casa de Misericórdia de Porto Alegre, Porto Alegre, RS – Brazil; 6 Brazil Escola Paulista de Medicina da Universidade Federal de São Paulo (EPM/UNIFESP), São Paulo, SP – Brazil; 7 Brazil Universidade de Pernambuco, Recife, PE – Brazil; 8 Brazil Universidade Católica de Pernambuco, Recife, PE – Brazil; 9 Brazil Faculdade de Ciências Médicas de Minas Gerais, Belo Horizonte, MG – Brazil; 10 Brazil Instituto de Hipertensão Arterial – Diretoria Clínica, Belo Horizonte, MG – Brazil; 11 Brazil Centro Universitário CESMAC – Centro de Pesquisas Clínicas Dr. Marco Mota, Maceió, AP – Brazil; 12 Brazil Universidade de São Paulo Faculdade de Medicina, São Paulo, SP – Brazil; 13 USA Duke University Hospital, Durham, North Carolina – USA; 14 Spain Hypertension and Vascular Risk Unit. Hospital Clinic. University of Barcelona, Barcelona – Spain

**Keywords:** Hypertension, Race Factors, Population Characteristics, Antihypertensive Agents

## Abstract

**Background:**

The prevalence of hypertension (HTN) is high in ethnic communities, particularly among Afro-descendant adults in the United States. Moreover, there is a lack of studies addressing this issue in the Brazilian Afro-descendant population.

**Objective:**

To analyze blood pressure control and the use of antihypertensive medications among Afro-descendant and non-Afro-descendant Brazilians.

**Methods:**

This cross-sectional study evaluated data from the First Brazilian Registry of Hypertension, which included individuals over 18 years of age, self-identified as Afro-descendant or non-Afro-descendant, with HTN for at least four weeks or using antihypertensive medication. Comparisons were performed using t-tests or the Mann-Whitney test. A significance level of p < 0.05 was adopted.

**Results:**

A total of 2,643 participants were included, of whom 82.8% were non–Afro-descendant and 17.1% were Afro-descendant. The rates of uncontrolled HTN were 44.68% among non–Afro-descendants and 54.64% among Afro-descendants. Median values of systolic blood pressure (SBP), diastolic blood pressure (DBP), and body mass index (BMI) were higher in Afro-descendants compared to non–Afro-descendants (p < .001). The distribution of antihypertensive medication classes varied between the populations. No significant differences in HTN control were observed between beta-blocker users in the Afro-descendant and non–Afro-descendant groups, even when stratified by sex.

**Conclusion:**

The Afro-descendant population in Brazil demonstrated a higher prevalence of uncontrolled HTN and higher SBP, DBP, and BMI values compared to the non–Afro-descendant population. The choice of antihypertensive medications differed between groups, with thiazides more commonly prescribed for Afro-descendants and ARBs for non–Afro-descendants. However, no significant differences in blood pressure control were observed between groups using beta-blockers, regardless of sex.

## Introduction

Hypertension (HTN) is the main modifiable risk factor for cardiovascular diseases (CVD) worldwide and remains the leading cause of death in the Americas.^1^In the United States, ethnic communities such as Afro-descendants, Hispanics, and Asians tend to develop HTN earlier and have lower blood pressure (BP) control rates compared to non-Hispanic whites.^2^The concept of race is widely debated and, in epidemiological studies, may obscure the true causes of health disparities between racial groups (diverting attention from socioeconomic and environmental factors), which contributes to the lower HTN control rates observed in these ethnic communities.^3 , 4^

Globally, in 2019, HTN diagnosis rates were 59% among women and 49% among men, while treatment rates were 47% in women and 38% in men.^5^Over the past four decades, global HTN patterns have shifted: prevalence has decreased in high-income countries while simultaneously increasing in low-income nations in South Asia and Sub-Saharan Africa. Meanwhile, the average number of cases per year has remained unchanged in Central and Eastern Europe.^6^

The prevalence of HTN is significant in ethnic communities, especially among Afro-descendant adults. The mortality rate associated with HTN among men and women of African descent is approximately twice as high compared to other racial/ethnic groups. The origins of these disparities are multifaceted and not fully elucidated.^2^Maintaining HTN control remains a persistent challenge in both clinical practice and public health.^7^

Despite the existence of socioeconomic and racial health disparities in Brazil, the implications of segregation on health outcomes have not yet been adequately analyzed in this context. This makes Brazil — with its similarities and differences compared to the United States — a particularly relevant setting for investigations in this field.^8^

In Brazil, the fusion of Afro-descendants and mixed-race groups forms a broad population category known as “ *Negra* ,” with estimates suggesting that the number of people with African ancestry exceeds the simple combination of Afro-descendant and mixed-race groups.^9^Currently, 55.5% of the Brazilian population identifies as Black, with self-declared Afro-descendants representing 20.7 million (10.3%) and mixed-race individuals 92.1 million (45.3%). Whites total 88.3 million (43.5%), Indigenous people 1.7 million (0.6%), and Asians 850,000 (0.4%).^10^

Considering that a significant portion of the Brazilian population has African ancestry, and that reduced control of HTN is observed specifically within this ethnic community—highlighting the challenges faced by the State—understanding such disparities can contribute to the development of fairer public health policies and enhance the effectiveness of clinical care for these vulnerable populations.^11^

## Methods

This cross-sectional study used data from the First Brazilian Registry of Hypertension (1RBH),^12^which was approved by the Ethics Committee on February 17, 2014, under protocol number 532.146. The 1RBH was a multicenter study that analyzed patients diagnosed with HTN. It included participants from all regions of Brazil, from public, private, and mixed healthcare services. Data collection occurred between June 2013 and October 2015.

The registry was conducted in strict accordance with national and international guidelines, including the Declaration of Helsinki, CNS Resolution 196/96 and its supplements (CNS/MS), the ICH Good Clinical Practice Guidelines (1996), the Americas Document (2005), and Resolution 466/2012. Each clinical research center submitted the study protocol, the Informed Consent Form (ICF), and all relevant documentation to its institution’s Research Ethics Committee (REC) for review and approval before initiating any procedures included in the registry.

After obtaining informed consent, medical records were reviewed, and participants were interviewed at each center by trained researchers to complete an electronic case report form (eCRF).

The analysis of the absolute frequency and percentage distribution of antihypertensive drug classes used by the population was conducted using the following categories: angiotensin receptor blockers (ARBs), thiazide diuretics, beta-blockers (BBs), angiotensin-converting enzyme inhibitors (ACE inhibitors), calcium channel blockers (CCBs), and loop diuretics. Individuals who self-identified as Black were classified as Afro-descendants, while those who self-identified as White, Brown (mixed-race), or Asian were classified as non-Afro-descendants.

Following the guideline recommendations at the time of the study, the mean of two peripheral BP measurements taken during the initial visit was used to assess BP control in groups. Participants were classified as having uncontrolled BP if their systolic BP (SBP) was ≥ 140 mmHg, diastolic BP (DBP) was ≥ 90 mmHg, or both. Those with controlled BP had SBP < 140 mmHg and DBP < 90 mmHg.

Inclusion criteria were: signing the ICF, being at least 18 years old, and having a confirmed diagnosis of HTN for at least four weeks, with SBP ≥ 140 mmHg and/or DBP ≥ 90 mmHg measured while seated,^12^or current use of antihypertensive medication, in addition to regular enrollment in the participating center/institution.

Exclusion criteria included: the presence of renal failure requiring dialysis, hospitalization at the time of inclusion or within the previous 30 days, hemodynamic instability requiring vasoactive drugs within the last 30 days, heart failure classified as functional class III or IV, pregnancy and/or breastfeeding, severe liver disease, psychiatric disorders preventing protocol adherence, a history of stroke or myocardial infarction within 30 days prior to study inclusion, severe illnesses as assessed by the investigator, and cancers with a survival prognosis of less than one year.

### Statistical analysis

Continuous variables were described using mean and standard deviation, or median (interquartile range), depending on data normality, which was tested using the Shapiro-Wilk test. Categorical variables were presented as counts and percentages. Statistical analyses were conducted in predefined subgroups, with p-values < 0.05 considered statistically significant. Comparative analyses were performed using the Mann–Whitney test when p < 0.05 and the t-test when p > 0.05. For binary logistic regression analysis, a 95% confidence interval was applied. In this context, uncontrolled BP, non-use of BBs, and female sex were set as reference level 1 to assess whether the use of BBs could be associated with better BP control. Statistical analyses were performed using Jamovi software, version 2.3.

## Results

### Patients’ characteristics

The study included 2,643 participants from 45 research centers across all regions of Brazil, of which 2,191 (82.9%) self-identified as non-Afro-descendant and 452 (17.1%) self-identified as Afro-descendant. Patients were treated in public (46.7%), private (31.1%), and mixed (22.2%) healthcare facilities. Detailed information on age, body mass index (BMI), waist circumference (WC), and sex for the general population and the non-Afro-descendant and Afro-descendant groups is available in Table 1 .

**Table 1:** – Characteristics of the patients included in the study between 2013-2015

Description	n	Non-afro-descendant	Afro-descendant	p
Self-Identified Ethnicity	2643	2191 (82.9%)	452 (17.1%)	
Sex, female	1472 (55.6%)	1212 (55.3%)	260 (57.5%)	0.390
Age, years	62.0 (54.1 - 69.4)	61.9 (54.1 - 69.4)	62.5 (54.2 - 69.7)	0.922
WC, cm	98.0 (90.0 - 107)	98.3 (90.0 - 107)	98.0 (91.0 - 106)	0.788
BMI, Kg/m^2^	28.7 (25.6 - 32.0)	28.4 (25.5 - 31.9)	29.4 (26.0 - 32.9)	0.002
HTN, duration years	10 (4 - 20)	10 (5 - 20)	10 (5 - 20)	0.044
HTN treatment, years	10 (4 - 17)	10 (4 - 17)	10 (5 - 20)	0.072
Diabetes Mellitus (DM)	784 (29.66%)	656 (29.9%)	128 (28.3%)	0.492
DM duration, years	5.1 (3 - 14)	7 (3 - 15)	6 (2 -10)	0.007
Alcohol consumption	197 (7.45%)	153 (6.9%)	44 (9.7%)	0.043
Smoking	165 (6.24%)	134 (6.1%)	31 (6.8%)	0.552

DM: Diabetes Mellitus; HTN: Hypertension; BMI: Body Mass Index; WC: Waist circumference. Source: developed by the author.

### Peripheral blood pressure values

Peripheral systolic and diastolic BP values were higher among Afro-descendant individuals compared to non-Afro-descendant individuals ( Table 2 ).

**Table 2:** – Comparative analysis of peripheral BP values in Brazilian hypertensive non-Afro-descendant and Afro-descendant populations, included in the study between 2013-2015

Blood pressure values	n (Non-Afro-descendant and Afro-descendant)	Non-Afro-descendant	Afro-descendant	p
SBP	2191; 452	132 (120–150)	140 (129–151^)^	< 0.001*
DBP	2191; 452	80.0 (73.5–90.0)	85.5 (80.0–93.0)	< 0.001*

* Mann-Whitney test, if p < 0.05. DBP (Diastolic blood pressure measured during the initial visit); SBP (Systolic blood pressure measured during the initial visit); SBP and DBP values are given in mmHg. Source: developed by the author.

Evaluation of blood pressure control in Brazilian hypertensive non-Afro-descendant and Afro-descendant populations

The Afro-descendant population showed a higher percentage of uncontrolled BP compared to the non-Afro-descendant population ( Figure 1 e Central Illustration ).

**Figure 1:** – Percentage of non-Afro-descendant and Afro-descendant individuals with controlled and uncontrolled blood pressure, included in the study between 2013 and 2015.

Antihypertensive medications used by hypertensive Afro-descendant and non-Afro-descendant populations in Brazil

Proportionally, the Afro-descendant population used a greater number of classes of antihypertensive medications than the non-Afro-descendant population ( Table 3 ).

**Table 3:** – Distribution of the absolute frequency and percentage of antihypertensive medication classes used by non-Black and Black Brazilian populations with hypertension, included in the study between 2013 and 2015

Number of classes *	Non-afro-descendant (n = 2,191)	Afro-descendant (n = 452)	p
0	109 (5.00%)	16 (3.50%)	0.967*
1	478 (21.80%)	115 (25.40%)	
2	793 (36.20%)	133 (29.40%)	
3	550 (25.10%)	120 (26.50%)	
4	204 (9.30%)	47 (10.40%)	
5	46 (2.10%)	17 (3.80%)	
6	11 (0.50%)	4 (0.90%)	

* Mann-Whitney test, if p < 0.05.

Classes of antihypertensive medications used by hypertensive individuals in Brazil, distinguishing between non-Afro-descendant and Afro-descendant populations

The percentage distribution of the first three classes of antihypertensive medications differed between the non-Afro-descendants and Afro-descendants groups, becoming similar only from the fourth class ( Table 4 ).

**Table 4:** – Descending order of the percentage of antihypertensive medication classes used by hypertensive Brazilian populations, non-Afro-descendant and Afro-descendant, included in the study between 2013 and 2015

Order	Non-afro-descendant (%) (n = 2,191)	Afro-descendant (%) (n = 452)
1º	47.6% (ARB)	52% (Thiazides)
2º	47.3% (Thiazides)	38.3% (Beta Blockers.)
3º	42.4% (Beta Blockers.)	38.1% (ARB)
4º	33.5% (ACE inhibitors))	35.6% (ACE inhibitors))
5º	28.1% (CCBs)	35.0% (CCBs)
6º	10.6% (loop diuretics)	16.6% (loop diuretics)
7º	7.4% (Spironolactone)	9.7% (Spironolactone)

ARB: angiotensin receptor blockers, ACE inhibitors: angiotensin-converting enzyme inhibitors, CCBs: calcium channel blockers, Thiazides: thiazide diuretics

Association between blood pressure control and the use of beta-blockers in hypertensive Brazilian afro-descendants and non-afro-descendants individuals and sex

No significant differences were found in BP control were found between Afro-descendant and non-afro-descendant groups using BBs, regardless of sex ( Tables 5 and 6 ).

**Table 5:** – Binary logistic regression analyses showing the odds ratio (OR) and 95% confidence interval (CI) for controlled vs. uncontrolled BP profiles, and for female and male individuals using beta-blockers in the hypertensive Black Brazilian population included in the study between 2013 and 2015

Independent Variables	OR	95% CI	p
Intercept	0.61	0.44 – 0.84	0.003
Beta-blocker			
Uses	1.04	0.70 - 1.53	0.840
Does not use	1		
Sex			
Male	1.25	0.85 – 1.83	0.254
Female	1		

Estimates represent the Log Odds of “BP profile = Controlled” vs. “BP profile = Uncontrolled”.

**Table 6:** – Binary logistic regression analyses showing the odds ratio (OR) and 95% confidence interval (CI) for controlled vs. uncontrolled blood pressure profiles, and for female and male individuals using beta-blockers in the hypertensive non-Black Brazilian population included in the study between 2013 and 2015

Independent Variables	OR	95% CI	p
Intercept	1.16	1.00 – 1.36	0.039
Beta-blocker			
Uses	0.93	0.78 – 1.11	0.433
Does not use	1		
Sex			
Male	0.87	0.74 – 1.04	0.133
Female	1		

The estimates represent the Log of the Odds for “BP profile = Controlled” vs. “BP profile = Uncontrolled”.

## Discussion

This study shows that the prevalence of uncontrolled HTN was higher among Brazilian Afro-descendant individuals, who also presented higher values of SBP, DBP, and BMI compared to the non-Afro-descendant population. We observed differences in the selection of antihypertensive medications prescribed by physicians, with greater use of thiazide diuretics among Afro-descendant individuals. However, among those also treated with BBs, no significant differences in HT control were identified between Afro-descendant and non-Afro-descendant groups, regardless of sex.

According to the latest Brazilian census, self-declared Afro-descendant individuals represent 10.2% of the Brazilian population,^10^which reinforces the relevance of our study, as we achieved 17.1% representativeness in the sample.

A recent study conducted in Brazil revealed that the likelihood of developing arterial HTN increased with age (particularly among Afro-descendant individuals) and was lower in the Northern region of the country, regardless of age group or sex. Notably, a higher probability of developing HTN was observed among women with lower educational levels and a sedentary lifestyle, as well as among men living with their spouses and engaging in low levels of physical activity.^13^

Our study also analyzed the issue of alcoholism and, with statistical significance, corroborated the findings of other similar studies, which indicate that, in Brazil, the highest incidence rate of this condition was again observed among the Afro-descendant population. Between 2013 and 2019, prevalence increased among Afro-descendants (from 16.6% to 19.6%), mixed-race individuals (from 11.2% to 17.5%), and whites (from 12.4% to 16.0%), while it decreased among Asians (from 14.4% to 12.7%).^14^

Some studies have pointed to a higher prevalence of type 2 diabetes and glucose intolerance among Afro-descendant people compared to whites.^15 , 16^Additionally, a Brazilian study showed that obese dark-skinned women demonstrated a higher association with diabetes and glucose intolerance, regardless of age and BMI.^17^

Interestingly, our data revealed an almost identical prevalence of diabetes between the populations; however, the non-Afro-descendant population exhibited a longer time to diagnosis. This may suggest that easier access to healthcare services among the non-Afro-descendant individuals favored earlier diagnosis and more effective disease management, which, in turn, may have helped mitigate the harmful effects of diabetes on the progression of HTN.

Afro-descendant participants showed significantly higher median values of both SBP and DBP compared to non-Afro-descendants, highlighting a trend toward elevated BP in this group. This disparity may be associated with socioeconomic and behavioral factors, such as differences in access to healthcare services, living conditions, health education, and the influence of social determinants that disproportionately affect the Afro-descendant population, contributing to less effective HTN control.

In the ELSA-Brasil cohort study, when analyzing the incidence of HTN by sex and race, Afro-descendant men showed the highest incidence (59.4 per 1,000 person-years), while White women had the lowest incidence (30.5 per 1,000 person-years). After adjusting for age and family history, the incidence rate remained significantly higher among Afro-descendant men (2.25; 95% CI, 1.65–3.08), followed by mixed-race men, Afro-descendant women, mixed-race women, and White men, compared to White women.^18^

Other studies have also demonstrated higher systolic and DBP levels among Afro-descendant populations compared to non-Afro-descendants.^19 - 22^Additionally, genetic research has shown the role of biological factors in the higher prevalence of HTN in populations of African descent.^23^However, genetic factors appear to exert less influence on the Brazilian Afro-descendant hypertensive population, considering that the effect of socioeconomic conditions on HTN management has been more evident.

Recent studies on variations in BP behavior between non-Afro-descendant and Afro-descendant populations have raised questions about the need to establish race-specific BP thresholds, which may improve risk estimation and optimize HTN management with the aim of reducing ethnic disparities.^22^In Brazil, the hypertensive Afro-descendant population showed a 9.76% lower control rate compared to the non-Afro-descendant population. This difference raises important questions about the factors that may contribute to the discrepancy in HTN control.

A study conducted in the United States, using similar diagnostic criteria for HTN (SBP ≥ 140 mmHg and/or DBP ≥ 90 mmHg), assessed a sample of 213,836 patients. The average age of participants was 63.1 years, with 55.5% women and 70.8% White. The study found that, without stratification by self-declared race, 29.7% of patients had uncontrolled HTN.^24^

Another study conducted in London investigated disparities in treatment and BP control in a large cohort of adult patients with HTN (n = 156,290). The study revealed that the Afro-descendant ethnic group had a lower likelihood of achieving BP control compared to the White ethnic group, concluding that Afro-descendant individuals and younger patients are less likely to attain HTN control.^25^

A study in the U.S. involving more than 700,000 patients reported annual rates of uncontrolled HTN ranging from 21.2% to 24.2%, with this variation being more common among men. African Americans had the highest rate (31.3%), while White men recorded 19.4%. Among women, African Americans showed 28.6% uncontrolled HTN, compared to 19.2% among White women.^26^

In parallel, a global study evaluating BP control showed that South Korea, Canada, and Iceland had the highest treatment and control rates, with more than 70% of cases receiving treatment and over 50% achieving control. Similarly, significant advances were observed in high-income nations and in some recently elevated upper-middle-income countries, including Costa Rica, Taiwan, Kazakhstan, South Africa, Brazil, Chile, Turkey, and Iran.^1^

A study conducted in the United States investigated variations in BP control between Afro-descendant and White patients before and after the implementation of a quality improvement (QI) program. The study observed that disparities in BP control between Afro-descendant and White patients decreased but were not fully eliminated, even after the introduction of QI strategies aimed at reducing these disparities.^27^

The Brazilian National Hypertension Control Registry, when evaluating HTN through office measurements and residential BP monitoring, without stratification by race, revealed that BP control in offices among a sample of Brazilians was 56.3%. This percentage increased to 61% when BP was measured at home and was 46.4% when control was observed both in the office and at home.^28^

In the United States, BP control rates have declined over the past decade, particularly among racial and ethnic groups. Non-Hispanic African Americans had control rates 10% lower than their non-Hispanic White counterparts.^29^A recent analysis of HTN control rates by the National Health and Nutrition Examination Survey (NHANES) revealed that BP control rates were lower for Hispanic individuals (40%), non-Hispanic afro-descendant people (NH) (39%), and Asian Americans (38%), compared to non-Hispanic White individuals (49%).^30^

Afro-Americans in the United States have considerably higher prevalence rates and lower control rates of HA compared to White populations.^31 , 32^Moreover, the frequent underrepresentation of Afro-Americans in cardiovascular clinical trials limits the safe application of results from various studies in this specific population.^33^

Several studies have highlighted ethnic disparities in HTN control, particularly among African Americans and Afro-Caribbeans (especially African American men), and have demonstrated the urgent need for targeted interventions to effectively address such inconsistencies in HTN management.^24 - 26^

Although in our study the most common number of medication classes was similar in both groups, differences were observed in the proportions of use. For example, Afro-descendants more frequently used monotherapy (25.4%) compared to non-Afro-descendants (21.8%). In contrast, White individuals, proportionally, used two antihypertensive medications more often than Afro-descendants. Finally, the Afro-descendant population, proportionally, used three or more classes of medications more frequently than the non-Afro-descendant population.

In a prospective cohort of North American women (n = 3,302; aged 42 to 52 years), Afro-descendant women were also found to have a higher likelihood of using more than two classes of antihypertensive medications (OR, 1.95; 95% CI, 1.55–2.45) compared to White women.^34^

A U.S. study involving 23,825 adults aged ≥18 years, which investigated racial and ethnic disparities in antihypertensive medication use among hypertensive adults from 2011 to 2018, revealed that the Afro-descendant population was significantly more likely to receive combination therapy and single-tablet combination medications.^35^In our study, the Afro-descendant population, in terms of frequency and without a statistically significant difference in the overall comparative analysis, showed a greater proportion of individuals using monotherapy.

Our study revealed that among afro-descendant participants, thiazide diuretics were the most common choice (52%), followed by BBs and angiotensin II ARBs. Among non-afro-descendant participants, ARBs were the most prescribed, followed by thiazide diuretics and BBs.

A systematic review on medication prescribing patterns in the United States for non-afro-descendants individuals indicated that the most commonly prescribed antihypertensive medications as monotherapy for adults with HTN without comorbidities were ACE inhibitors or ARBs, followed by CCBs and BBs. The most prevalent dual combinations were thiazide diuretics with ACE inhibitors or ARBs, BB with CCB, and CCB with ACE inhibitors or ARBs.^36^Another review indicated that thiazide diuretics were identified as the most frequently prescribed antihypertensives as monotherapy, while ACE inhibitors or ARBs were the most prescribed in another study.^37^

The results of the ELSA-Brasil Study suggest that, in a sample of Brazilian adults using antihypertensive monotherapy, differences in BP control among various racial groups are not explained by the potentially lower efficacy of ACE inhibitors and ARBs in Afro-descendant individuals. It is emphasized that the Afro-descendant population in Brazil presents distinct characteristics, and recommendations should be adapted to the local context, indicating that the lower BP control observed in Afro-descendant individuals may be more closely related to social determinants than to the class of antihypertensive medication used.^38^

These results highlight the importance of considering ethnic and individual factors in prescribing antihypertensive medications, in addition to socioeconomic issues, as different ethnic groups may have varied responses to treatments.^39^Furthermore, it is evident that there are differences in the classes of medications prescribed in Brazil compared to the United States. In this study, we also explored the hypothesis that the use of BBs could be associated with better HTN control. However, we did not identify significant differences in HTN control between the two groups, regardless of sex, which led us to question of whether the use of BBs in combination may have a lesser effect on BP reduction compared to monotherapy in our Afro-descendant and non-Afro-descendant populations.

A meta-analysis evaluating 20 studies revealed that β-blockers, except for atenolol, are effective in lowering BP, both as an adjunct to monotherapy and as part of a combination anti-hypertensive therapy. No significant differences were found in the BP-lowering effects between combinations with and without BBs (SBP: -1.3 mm Hg [-5.8 to 3.2]; DBP: -0.3 mm Hg [-2.7 to 2.1]).^40^

In another study involving 11,860 participants, second- and third-generation BBs reduced average BP by 1.75 mmHg (95% confidence interval: 1.16–2.33; p < 0.001) across all participants analyzed, and by 1.93 mmHg (95% CI: 0.86–3.00; p < 0.001) specifically among hypertensive Africans.^41^Understanding why the use of BBs was not associated with improved BP control remains unclear. However, this may be related to the fact that many hypertensive individuals were using more than two classes of medications.

### Limitations

It is important to note that the analysis did not provide information on the underlying reasons for social, educational, economic, and behavioral disparities—such as access to healthcare services, treatment adherence, or genetic factors—which may limit the interpretation of the results.

## Conclusions

With regard to BP control, self-identified Afro-descendant Brazilians exhibited a higher proportion of uncontrolled HTN compared to non-Afro-descendant individuals. The Afro-descendant population also showed higher peripheral BP values than the non-Afro-descendant population. Differences were observed in the percentages of the most commonly used medications and in the distribution of drug classes. No significant associations were found in BP control among the groups using BBs, regardless of sex.
